# Computational Nuclear Oncology Toward Precision Radiopharmaceutical Therapies: Current Tools, Techniques, and Uncharted Territories

**DOI:** 10.2967/jnumed.124.267927

**Published:** 2025-04

**Authors:** Tahir Yusufaly, Emilie Roncali, Julia Brosch-Lenz, Carlos Uribe, Abhinav K. Jha, Geoffrey Currie, Joyita Dutta, Georges El-Fakhri, Helena McMeekin, Neeta Pandit-Taskar, Jazmin Schwartz, Kuangyu Shi, Lidia Strigari, Habib Zaidi, Babak Saboury, Arman Rahmim

**Affiliations:** 1 Division of Radiology and Radiological Sciences, Johns Hopkins School of Medicine, Baltimore, Maryland;; 2 Department of Biomedical Engineering, University of California Davis, Davis, California;; 3 Institute of Nuclear Medicine, Glen Burnie, Maryland;; 4 Department of Radiology, University of British Columbia, Vancouver, British Columbia, Canada;; 5 Department of Biomedical Engineering and Mallinckrodt Institute of Radiology, Washington University, St. Louis, Missouri;; 6 School of Dentistry and Medical Sciences, Charles Sturt University, Wagga Wagga, New South Wales, Australia;; 7 Department of Biomedical Engineering, University of Massachusetts, Amherst, Massachusetts;; 8 Department of Radiology and Biomedical Imaging, Yale University School of Medicine, New Haven, Connecticut;; 9 Hermes Medical Solutions, London, United Kingdom;; 10 Department of Radiology, Memorial Sloan Kettering Cancer Center, New York, New York;; 11 Department of Radiology, Weill Cornell Medical College, New York, New York;; 12 Department of Medical Physics, Memorial Sloan Kettering Cancer Center, New York, New York;; 13 Department of Nuclear Medicine, University of Bern, Bern, Switzerland;; 14 Department of Medical Physics, IRCCS Azienda Ospedaliero-Universitaria di Bologna, Bologna, Italy;; 15 Division of Nuclear Medicine and Molecular Imaging, Geneva University Hospital, Geneva, Switzerland;; 16 Department of Nuclear Medicine and Molecular Imaging, University Medical Center Groningen, University of Groningen, Groningen, Netherlands;; 17 United Theranostics, Bethesda, Maryland; and; 18 Department of Physics, University of British Columbia, Vancouver, British Columbia, Canada

**Keywords:** radiobiology, radionuclide therapy, artificial intelligence, computational nuclear oncology, dosimetry, theranostic digital twin

## Abstract

Radiopharmaceutical therapy (RPT), with its targeted delivery of cytotoxic ionizing radiation, demonstrates significant potential for treating a wide spectrum of malignancies, with particularly unique benefits for metastatic disease. There is an opportunity to optimize RPTs and enhance the precision of theranostics by moving beyond a one-size-fits-all approach and using patient-specific image-based dosimetry for personalized treatment planning. Such an approach, however, requires accurate methods and tools for the mathematic modeling and prediction of dose and clinical outcome. To this end, the SNMMI AI-Dosimetry Working Group is promoting the paradigm of computational nuclear oncology: mathematic models and computational tools describing the hierarchy of etiologic mechanisms involved in RPT dose response. This includes radiopharmacokinetics for image-based internal dosimetry and radiobiology for the mapping of dose response to clinical endpoints. The former area originates in pharmacotherapy, whereas the latter originates in radiotherapy. Accordingly, models and methods developed in these predecessor disciplines serve as a foundation on which to develop a repurposed set of tools more appropriate to RPT. Over the long term, this computational nuclear oncology framework also promises to facilitate widespread cross-fertilization of ideas between nuclear medicine and the greater mathematic and computational oncology communities.

Nuclear medicine is in a renaissance through radiopharmaceutical therapies (RPTs) that have demonstrated efficacy, particularly in treatment of advanced metastatic cancers (1–3). Radiopharmaceuticals are administered systemically and deliver radiation locally as molecules preferentially bind to cancer-specific targets.

Current labels of federally approved RPTs, however, follow a one-size-fits-all approach with fixed activities, numbers of cycles, and intercycle time intervals (4,5). This practice neglects patient-specific characteristics that modulate therapeutic response, including biokinetics, risk factors, functional organ reserves, and tumor heterogeneity (6–9). Consequently, the delivered doses to organs or tumor, and resulting outcomes, can vary significantly (10,11), sometimes by orders of magnitude (12,13).

Accordingly, there has been interest in personalizing RPT. Recently, road maps toward this goal have been proposed (7,14), with an emphasis on theranostic digital twins: virtual, patient-specific avatars parameterized with pretreatment diagnostic imaging and clinical input, simulated to prospectively predict dosimetry and outcomes and optimized to design individualized activity prescriptions and treatment plans, including iterative feedback and treatment adaptation between cycles, as shown in Figure 1. The development and implementation of theranostic digital twins require research advancements on many fronts, highlighted by a recent review (7). Examples (15,16) of problems that must be resolved include population-level initialization and patient-specific fine-tuning of model parameters; standardization and harmonization of heterogeneously collected data; and verification, validation, and uncertainty quantification.

**FIGURE 1.**
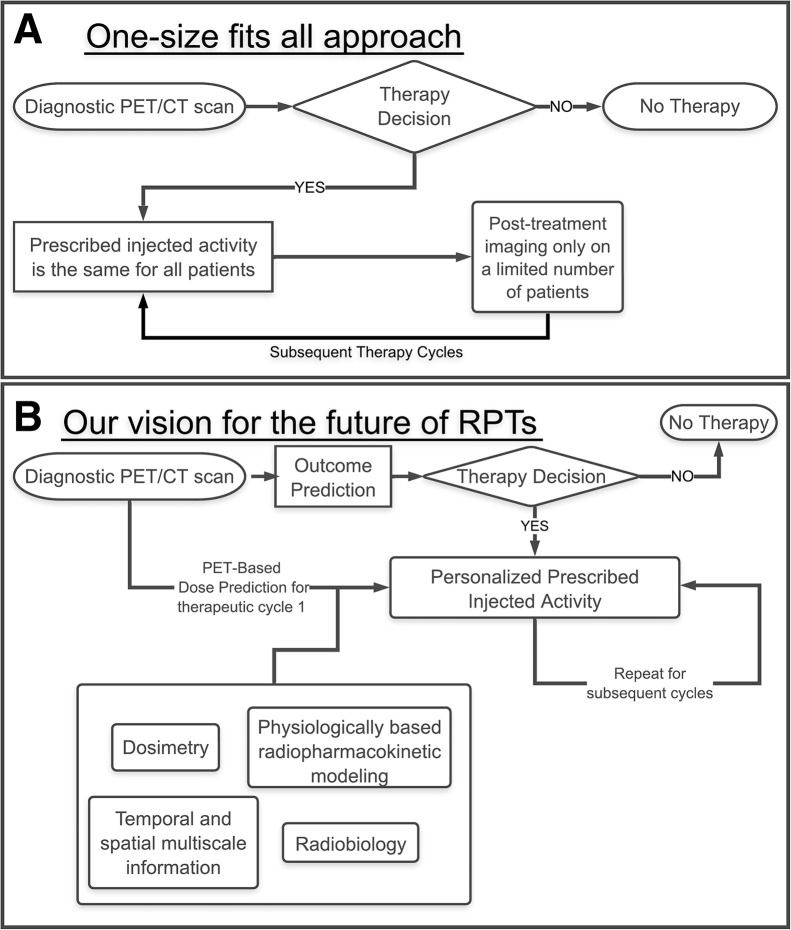
Diagram comparing present one-size-fits-all RPT approach (A) with vision of precision RPTs (B). Central to such a future is development of robust models that translate therapeutic inputs to clinical outputs, from which one can prescribe optimal protocols in cycle 1 and refine the process in later cycles to adaptively optimize treatment.

Central to all of these is the identification and selection of robust mathematic models mapping input treatment protocols to predicted clinical outcomes. Therapeutic response to RPT is driven by a combination of 2 ingredients: pharmacokinetics (17,18), which determines the agent biodistribution and resulting physical dose to tumors and organs, and radiation biology (19–21), which translates this spatiotemporal dose distribution to biologic effects, including efficacy and toxicity. These ingredients are relics of RPT’s historic relationship to 2 therapeutic paradigms: pharmacotherapy (for pharmacokinetics) and radiation therapy (for radiobiology). Both of these communities have independently developed mathematic formalisms and computational techniques tailored to their purposes. Thus, integration of methods from these 2 specialties is a promising place to start in identifying an appropriate set of tools for RPT.

However, it is important to realize that this hybridization also endows RPT with unique features that differentiate it from its predecessors. For example, whereas concepts from traditional pharmacokinetics carry over, in RPT we now have the additional feature that the site of action of the therapeutic agent (radiation) is not commensurate with the physiologic biodistribution of the pharmaceutical but also depends on physical characteristics of the emitted radiation tracks in relation to organ anatomy. In addition, the emission of radiation endows us with the unique opportunity to image delivery in real time. Likewise, even though biologic effects are driven by radiation, the radiobiology of external-beam radiation therapy (EBRT) is not directly transferable to RPT, because of differences in spatial and temporal heterogeneity between the 2 modalities. These originate, again, in physiology-mediated biokinetics, which needs to be estimated with nuclear medicine imaging in order to calibrate radiobiologic calculations.

If RPT can be considered related to the predecessor therapies of pharmacotherapy and radiation therapy, then we define computational nuclear oncology (CNO) as the combination of methods from these 2 respective communities, but again with distinct properties originating from nuclear medicine, as illustrated in Figure 2. With this in mind, the goal of the current paper is to review techniques available for mathematic and computational modeling in the predecessor disciplines, with an eye toward how various toolkits must to be adapted and integrated to deal with peculiarities of RPT.

**FIGURE 2.**
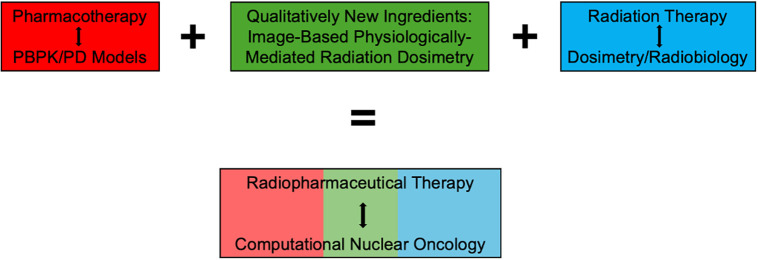
RPT is hybridization of 2 therapeutic paradigms, pharmacotherapy and radiation therapy, but with additional element of physiologically mediated delivery that can be imaged through nuclear medicine. Accordingly, CNO may be considered analogous hybrid of pharmacokinetic modeling and radiobiology-calibrated dosimetry, linked via intermediary of image-based internal dosimetry. PD = pharmacodynamics.

## REPURPOSING PHYSIOLOGICALLY BASED PHARMACOKINETICS (PBPK) INTO PHYSIOLOGICALLY BASED RADIOPHARMACOKINETICS (PBRPK) FOR PERSONALIZED RPT DOSIMETRY

Reliable modeling and prediction of pharmacokinetics is the first step toward clinical RPT dosimetry (22–25). The mathematic methods appropriate to this task are rooted in research originating from the drug discovery and development communities (26–28). Particularly relevant to RPT is PBPK modeling. PBPK models represent organs as individual compartments connected via the circulatory system, which facilitates tracer accumulation, transfer, and exchange through mechanisms such as perfusion or permeability constraints. Each organ is further broken down into subcompartments (e.g., interstitial, vascular, and cellular) linked by physical and biochemical pathways. The choice of compartments and level of detail included are specific to each drug or radiopharmaceutical, its biodistribution, and the targeted pathologic process. Reference tissue models, which obviate individual blood concentration calibrations, are also a viable approach.

PBPK models have a rich tradition in pharmacology and are now a routine tool for in silico drug discovery and development (26). The diffusion of PBPK into the RPT community is a more recent phenomenon (23,25) that has opened a wide array of exciting opportunities. It has also, however, introduced new challenges arising from the idiosyncrasies of radiopharmaceuticals.

Most notably, RPT delivers therapy nonlocally via the emission of radiation tracks, which propagate to distal targets throughout the body. Thus, PBPK models of the radioactive emitter must also be paired with internal dosimetry (29,30), which translates the biodistribution in different source organs to the resulting absorbed dose (AD) to biologic targets. Such calculations are intricate, necessitating the consideration of multiple factors (31), including the isotope’s decay scheme, including energy, emitted particle, and decay branching ratios; particle range and half-life; the material, typically biologic tissue, where decay occurs and energy is deposited; and the total number of disintegrations in the tissue. Approaches to internal dosimetry in RPT range from organ-level S values (32–34) to detailed 3-dimensional approaches, such as voxel S values (35–37) and Monte Carlo simulations (38–40). The inclusion of dosimetry distinguishes pharmacokinetic modeling in RPT from more conventional pharmaceuticals. Furthermore, in RPT, hot and cold pharmaceuticals interact though decay as well as through competition in binding to receptors (25,41), which distinguishes such modeling sufficiently to justify its own name: PBRPK.

In addition, radiopharmaceuticals also differ from more traditional agents by being traceable after administration via molecular imaging (42,43), which quantifies accumulation and clearance of the radiopharmaceutical in different tissues over time. Thus, RPT offers possibilities for real-time imaging and assessment of the therapeutic biodistribution via simultaneous emission of photons. Common techniques for this include conjugate-view γ-camera imaging, SPECT, and hybrid SPECT/planar imaging (44), with SPECT demonstrating superior accuracy (45,46). Furthermore, RPT also allows for prospective dose prediction in addition to retrospective dose assessment (47–49). By exploiting newly introduced high-sensitivity long-axial-field-of-view and total-body PET scanners (50–52), multiple-time-point and delayed pretherapy scans can be used to infer individual-patient time–activity curves for theranostic pairs (53). Notably, these recent innovations allow us, for the first time, to observe pharmacokinetic dynamics spanning multiple timescales simultaneously, including both conventional drug biodistribution and clearance operating over the course of several hours or days, along with more rapid uptake and washout processes that take place over shorter timespans of 1–2 h.

**FIGURE 3.**
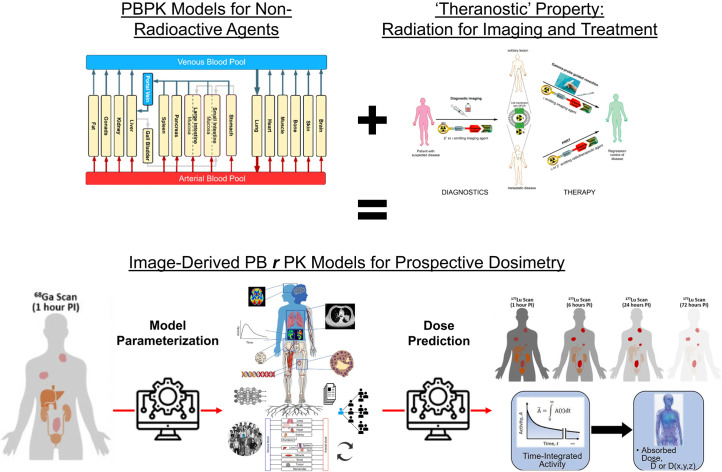
PBPK modeling and simulation from drug discovery and development communities can be applied to enable RPT dosimetry via theranostic principle, which enables estimation of patient-specific pharmacokinetics with pretherapy PET imaging, and use these models to help prospectively predict patient-specific therapeutic doses that can be verified with real-time SPECT imaging. PI = after injection. (PBPK figure adapted from (118), theranostics schematic adapted from (119), diagnostic PET and therapeutic SPECT cartoons adapted from (120), model-parameterized PBRPK network adapted from (8), and time-integrated activity–to–AD diagram adapted from (121).)

Collectively, these efforts will lead to a future CNO pipeline, illustrated in Figure 3, where pretherapy image-derived time–activity curves from the diagnostic can be used to extrapolate and parametrize a PBRPK model for the therapeutic. The resulting model can be used to prospectively predict, for a given choice of therapeutic protocol actions, the AD to tumors and normal organs. Paired with appropriate models for translating AD to clinical endpoints (to be discussed in the next section), clinicians can tweak protocol actions to optimize therapeutic efficacy.

Image-based PBRPK modeling and dosimetry have already been applied to several interesting questions. Examples include studies on the effects of vascular perfusion and receptor density on tumor delivery (54); the effects of ligand amount, affinity, and internalization on AD (55,56); the effects of time-point image sampling on quantitative measures (57); the effect of tumor volume on AD in kidneys and tumors (58); the prediction of therapeutic biodistribution from biokinetics, tumor volume, and glomerular filtration rate (59); and the impact of administration scheduling, including single versus multiple-bolus injections, on AD (25).

## RECALIBRATING EBRT RADIOBIOLOGY FOR MULTISCALE RPT DOSIMETRY

After image-based pharmacokinetic estimation of AD, the remaining step in the CNO pipeline is to translate this AD to clinical outcomes. In EBRT, this goal is achieved using the methods of radiobiology (60,61). Radiation kills cells by causing DNA damage through mechanisms that include direct ionization, free radical generation, and activation of secondary processes. Double-strand breaks are particularly harmful, leading to genetic information loss and incorrect rejoining, which are often lethal. Factors such as cell proliferation, differentiation, and DNA repair capabilities determine the radiosensitivity of a cell, which is also modified by dose rate, oxygenation, and linear energy transfer.

The most commonly used clinical model in EBRT for these effects is the linear-quadratic model (62), sometimes modified to incorporate factors such as incomplete repair of sublethal damage. This model serves as a springboard for deriving (63) the biologic effective dose, which may be thought of as a local calibration of the spatiotemporal dose distribution to the cells in a microscopic subvolume of the organ or tumor. The remaining step is to translate the overall distribution of the biologic effective dose across the structure into an overall tumor control probability or normal-tissue complication probability. Essential in this is consideration of the specific clinical endpoint and the extent to which the organ behaves serially (strong codependence of the functional subunits) versus parallelly (the functional subunits behaving mostly autonomously). The tumor control probability and normal-tissue complication probability serve as conceptual underpinnings (64,65) of the physical and dosimetric constraints built into modern EBRT treatment planning software, such as Varian RapidPlan (66) or Accuray RayStation (67).

A pressing challenge for RPT, however, is that the radiobiologic knowledge and assumptions built into EBRT treatment planning are specifically configured for that modality. Currently, with limited data, RPT often assumes EBRT-derived dose limits. Although in some cases (e.g., long-ranged β-emitting RPT with dose-rate biologic effective dose corrections) such approaches might be reasonable, it is generally agreed that the black-box application of EBRT paradigms hinders the long-term development of RPT and leads to suboptimal outcomes (68). Radiobiologic effects in RPT (20,21) arise from distinct patterns of particle type, energy, transport, range, and dose rate originating in pharmacokinetics, which shapes dose in a way that is fundamentally different from EBRT (69,70). As a result, the extent to which models and assumptions used in EBRT can be extrapolated remain unclear.

This is especially important for α-emitters because of the prevalence of microdosimetric fluctuations at the cellular and subcellular levels, phenomena that have no analog in photon EBRT. Relatedly, understanding differences in DNA repair, cell death, and cell cycle effects between the 2 modalities is essential. It is known that these processes are sensitive to microenvironmental variations, such as tumor hypoxia and vasculature or infiltration of immune cells and products. Given the differences in radiation delivery between RPT and EBRT, it is reasonable to assume that any radiation-induced alterations to this microenvironment would also differ. Nuclear medicine, incidentally, is well suited to imaging such changes in order to quantify biologic effectiveness.

**FIGURE 4.**
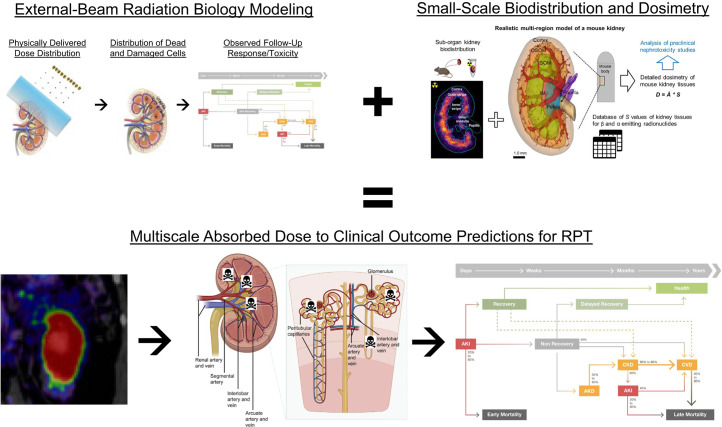
Concepts, tools, and models of external-beam radiobiology, from which much of modern EBRT treatment planning is based, must be modified for RPT because of physiologically mediated small-scale biodistribution and dosimetry, which modify radiation response via series of complex and incompletely understood mechanisms. RPT-adapted radiobiologic modeling must ultimately be paired with suitably estimated multiscale extrapolation to enable robust end-to-end prediction of clinical outcome, illustrated here for the example of acute kidney injury. AKD = acute kidney disease; AKI = acute kidney injury; CKD = chronic kidney disease; CVD = cardiovascular disease; IM = inner medulla; ISOM = inner strip of outer medulla; OSOM = outer strip of outer medulla. (Multiscale anatomy diagrams adapted from (122), acute injury dynamic function schematic adapted from (123), PET/CT adapted from (124), and multiscale dosimetry figure adapted from (125).)

Accordingly, developing robust methodologies to adapt EBRT-based radiobiology to RPT, or create new RPT-specific models de novo, is imperative. A good starting point to this end, illustrated in Figure 4, is to interface quantitative radiobiology with PBRPK-derived inferences of the microscale dose distributions, with all biologic effective dose, tumor control probability, and normal-tissue complication probability models adjusted (71–73) to correct for the differences in RPT and EBRT. This interfacing, however, is made challenging by the need to model and the coupling of dosimetric processes across multiple scales (74–76). Current image-derived PBRPK dosimetry yields information on mean organ or tumor ADs or on ADs per voxel of several-millimeter size. On the other hand, radiobiologic modeling starts with DNA damage and cell death in tissues, processes that occur at the microscale and nanoscale.

Various approaches exist for extrapolating observable organ-level energies to doses at the suborgan (77,78), voxel (79), cellular (73), and molecular (80,81) levels. Incorporating these subresolution nonuniformities into the radiobiologic simulations will be important for reliable outcome prediction. These subgrid extrapolation schemes are typically measured in vitro using preclinical animal models (82). Examples of common software packages used in performing such tasks include GEANT4 (83) and its extensions (81,84), MCNP (85,86), FLUKA (87), PENELOPE (88,89), and various components in the MIRDsoft platform (90).

However, in their present form, these tools are mostly of academic interest, and additional work is critically needed to bridge them with clinical tools (44), examples including Rapid (91) or Voximetry (92). It is encouraging that this area has recently seen an increase in activity, particularly in Europe (21,93–95). Building on this momentum, future work should prioritize preclinical investigations of small-scale radiopharmaceutical distribution, radiation-induced DNA damage and repair, and the coupling of macroscopic imaging with these microscopic data. A noteworthy example to this end built a cellular-level dosimetry model for targeted α-therapy with Monte Carlo simulations, which was experimentally validated in vitro for ^212^Pb (96).

## FORWARD-LOOKING VISION: CNO AS A BASIS FOR CROSS-SPECIALTY DIGITAL TWINS

The previous sections have reviewed computational predecessors of CNO, describing how they must be adapted for RPT. In so doing, we have highlighted how prospective image-based PBRPK dosimetry can calculate tumor and organ AD and how radiobiologic calculations supplemented by small-scale subgrid dosimetry models can translate AD into observed clinical effects for treatment optimization. It is encouraging to see the progress that has already been made in each of these pieces. The natural next step is to sync the pieces together and interface the models with real-world clinical data, including validation, verification, and uncertainty quantification (97,98). The timing for these efforts is ripe, given the recent calls in the RPT community (68,99) for more robust data collection and standardized reporting of imaging, dosimetry, and outcomes. The myriad technical and logistic challenges that must be addressed to this end have been reviewed elsewhere (3,100).

We would like to draw attention to an intriguing, but often neglected, ancillary benefit of theranostic digital twins and the underlying CNO framework discussed herein. In this review, we have emphasized links between modeling paradigms from the communities of pharmacotherapy and radiation therapy to RPT. The resulting tools can serve as a powerful unifying framework for cross-institutional data sharing and model building across nuclear medicine clinics. However, in addition to facilitating intramodality sharing within the RPT community, CNO is also a natural language for facilitating intermodality sharing and cross-disciplinary fertilization with other medical specialties. The desire and push for data-driven modeling and digital twin personalization is not unique to nuclear medicine (101–103), and the medical subspecialities associated with the predecessor disciplines of CNO—medical oncology for pharmacotherapy (104,105) and radiation oncology for radiation therapy (106,107)—have also seen recent enthusiasm for digital twins. By emphasizing the relationship of RPT to these specialties, CNO will allow the nuclear medicine community to pursue appropriate stewardship of RPTs while also being able to synergistically interact with medical “relatives.”

This will also serve as a gateway for more regular cross-fertilization across the greater computational and mathematic oncology community. The application of mathematics to cancer biology and treatment response has a rich history, and recent years have seen an explosive number of breakthroughs in the field. Examples of such progress include ordinary differential equation models of tumor growth and response (108), partial differential equation models of tissue reorganization and metastasis (109), agent-based models of intercellular interactions and behavior (110), stochastic models of tumor ecoevolutionary extinction (111), and game-theoretic optimization of combination and adaptive therapies (112). Clearly, nuclear medicine and the RPT community have much to gain by synergizing with the state-of-the-art mathematic and computational modeling tools. CNO, as such, will facilitate the interdisciplinary communications necessary to realizing these gains.

## CONCLUDING THOUGHTS

In this article, we have introduced CNO as a computational modeling approach that will ultimately enable personalization of RPTs via theranostic digital twins. Our exposition highlighted how RPT includes elements of both pharmacotherapy and radiation therapy but maintains its unique identity. These interdisciplinary origins, moreover, will help guide the nuclear medicine community in further adapting and integrating computational tools from other communities into the CNO toolkit.

As a final comment, it is irresistible to point out analogies and similarities between this discussion and the machine learning areas of large language models, foundation models, and artificial general intelligence (113,114). Interest in these fields is predicated on the idea that models trained on specific data modalities for particular tasks can be repurposed, via transfer learning and other methods (115), for application to new data modalities and tasks. Identifying which features are task- and modality-specific, and determining what can and cannot be transferred, is very much akin to the way CNO keeps certain elements from its predecessors while discarding others. One of the important lessons learned throughout the larger oncology community is that there is no magic bullet for cancer (116,117). A truly long-term approach to treatment will require a multipronged strategy as flexible and adaptive as the tumor being treated (112). Such visions are exciting to dream about but challenging to operationalize. We believe that CNO, and how it connects RPT to pharmacotherapy and radiation therapy while emphasizing its distinctiveness, will serve as an example to the medical community on how to practically realize these goals.

## DISCLOSURE

Arman Rahmim and Carlos Uribe are cofounders of Ascinta Technologies Inc. No other potential conflict of interest relevant to this article was reported.
